# Benzophenone-3 Impairs Autophagy, Alters Epigenetic Status, and Disrupts Retinoid X Receptor Signaling in Apoptotic Neuronal Cells

**DOI:** 10.1007/s12035-017-0704-2

**Published:** 2017-08-16

**Authors:** Agnieszka Wnuk, Joanna Rzemieniec, Władysław Lasoń, Wojciech Krzeptowski, Małgorzata Kajta

**Affiliations:** 1 Department of Experimental Neuroendocrinology, Institute of Pharmacology, Polish Academy of Sciences, Smetna Street 12, 31-343 Krakow, Poland; 2 Department of Cell Biology and Imaging, Institute of Zoology, Jagiellonian University, Gronostajowa Street 9, 30-387 Krakow, Poland

**Keywords:** Benzophenone-3, BP-3, Retinoid X receptors, RXR, Primary neuronal cell cultures, Autophagy

## Abstract

Benzophenone-3 (BP-3) is the most widely used compound among UV filters for the prevention of photodegradation. Population studies have demonstrated that it penetrates through the skin and crosses the blood-brain barrier. However, little is known about the impact of BP-3 on the nervous system and its possible adverse effects on the developing brain. We demonstrated that the neurotoxic effects of BP-3 were accompanied by the induction of apoptosis, as evidenced by apoptosis-related caspase-3 activation and apoptotic body formation as well as the inhibition of autophagy, as determined by the downregulation of autophagy-related genes, decreased autophagosome formation, and reduced LC3B-to-LC3A ratio. In this study, we showed for the first time that the BP-3-induced apoptosis of neuronal cells is mediated via the stimulation of RXRα signaling and the attenuation of RXRβ/RXRγ signaling, as demonstrated using selective antagonist and specific siRNAs as well as by measuring the mRNA and protein expression levels of the receptors. This study also demonstrated that environmentally relevant concentrations of BP-3 were able to inhibit autophagy and disrupt the epigenetic status of neuronal cells, as evidenced by the inhibition of global DNA methylation as well as the reduction of histone deacetylases and histone acetyl transferases activity, which may increase the risks of neurodevelopmental abnormalities and/or neural degenerations.

## Introduction

Because of public anxiety about skin cancer caused by ultraviolet light (UV), production and consumption of sunscreen products are increasing. Nowadays, over 10,000 t of UV filters are produced annually for the global market [1]. Chemical UV filters are generally used as a mixture since none of the compounds used individually get sufficient protection against UV.

Among the filters, benzophenones (BPs) are the primary ingredients in the organic UV filter family. Benzophenone-3 (2-hydroxy-4-methoxybenzophenone, oxybenzone, 2OH-4 MeO-BP or BP-3) is the most widely used compound among BPs for the skin prevention against photodegradation [2]. Human studies have demonstrated that after topical application, BP-3 is absorbed through the skin, partially metabolized, and is excreted in the urine. BP-3 was detected in almost all (80–96%) urine samples collected from the general population in the USA [3]; pregnant women in France [4, 5]; and Danish mothers and their children, adolescents, young men, and pregnant women [6, 7]. BP-3 can be detected in the serum and urine of adult volunteers shortly after dermal application, proving that it can pass through the skin into the body [8]. It has been estimated that 10% of the applied dermal BP-3 penetrates skin into systemic circulation [9].

It is extremely disturbing that BP-3 has been detected in a large proportion of milk samples indicating that breastfed babies are exposed to BP-3 [10]. A recent study showed that maternal exposure to BP-3 is strongly associated with the onset of Hirschsprung’s disease in offspring [11]. However, data on the effects of BP-3 on the nervous system are scarce. Especially little is known about the impact of BP-3 on individual receptors that are strongly associated with brain development such as retinoid X receptors. The only data on the apoptotic and neurotoxic effects of BP-3 on the neural cells come from SH-SY5Y neuroblastoma cells and our recently published original paper [12, 13]. BP-3 has been reported to act as endocrine disrupting chemicals (EDCs). At present, it is only known that BP-3 can weakly agonize estrogen signaling and strongly antagonize androgen-related pathways [14–16]. An association between BP-3 exposure and the estrogen-related disease endometriosis has been found [17]. Recently, retinoid X receptors have been postulated to be a target for EDCs.

The retinoid X receptor (RXR) is a type of nuclear receptor family that is encoded by three genes: *RXRα*, *RXRβ*, and *RXRγ* [18]. RXRs heterodimerize with one-third of the 48 human nuclear receptor superfamily members [19]. For most of them, RXR is an obligatory partner for DNA binding and transcriptional regulation. In addition, RXRα is able to form homodimers and homotetramers, which is suggestive of the self-regulation of specific RXRα signaling pathways [20]. The diversity of RXRs suggests that they play critical roles in a wide range of cellular pathways. Recent studies have shown the prominence of RXR signaling in developing innervation and myelination in health and disease of the central nervous system [21]. Current studies in our research group have shown the involvement of RXRs in the effects of EDCs (specifically the pesticide dichlorodiphenyldichloroethylene (DDE) and nonylphenol) [22–24].

One of the most important ways of regulating gene expression is the remodeling of chromatin, including post-translational modifications of histones and DNA methylation. It has been postulated that low doses of EDCs may cause epigenetic changes, such as the incomplete methylation of specific gene regions in the young brain [25, 26]. Histone post-translational modifications include the most studied modifications—the acetylation of histones by histone acetyl transferases (HATs) and the removal of acetyl groups from histones by histone deacetylases (HDACs). These processes play important roles in cognition as well as psychiatric and neurologic diseases such as Alzheimer's disease, Huntington’s disease, traumatic brain injury, post-traumatic stress disorder, stress, depression, and addiction [27].

Autophagy is a process that is mainly responsible for eliminating the cells or keeping them alive, even in conditions deprived of trophic factors. Autophagy is postulated to play a housekeeping role in removing abnormal proteins or clearing damaged organelles. The formation of autophagosomes depends on several core Atg proteins, such as the following: ULK1 complex, Beclin1:Vps34/Atg14L complex, and LC3 conjugation systems. During the process of autophagy, LC3 protein is cleaved by Atg4 to LC3A which next is modified by ubiquitin-like systems to produce LC3B. Thus, LC3A and LC3B are present in autophagosomes; both the ratio of LC3B to LC3A and the amount of LC3B only can be used to estimate the level of autophagy. Recent studies have proposed that generally autophagy is a survival mechanism, although its dysregulation may lead to non-apoptotic cell death [28].

The present study aimed to investigate the neurotoxic and apoptotic effects of BP-3 and the impact of this chemical on the expression and function of RXRs, including RXRα, RXRβ, and RXRγ. Neurotoxicity was estimated by measuring lactate dehydrogenase (LDH) release, which was complemented by an assessment of caspase-3 activity. These data were supported by Hoechst 33342/calcein acetoxymethyl (AM) staining, which allowed for the visualization of apoptotic nuclei and cell survival. The involvement of RXRs in the actions of BP-3 was verified using selective antagonist and agonist as well as specific siRNAs. The levels of receptor mRNAs and proteins were measured with qPCR, western blot, and enzyme-linked immunosorbent assay (ELISA), and the cellular distributions of the receptors were demonstrated using a confocal microscope. The process of autophagy was assessed by measuring the expression of autophagy-specific genes using microarray analysis and autophagosome detection, and the concentrations of autophagy-selective proteins were measured by ELISAs. Results regarding epigenetic modifications such as histone post-translational modifications and DNA methylation were complemented by an assessment of HAT and HDAC activity and the measurement of global DNA methylation.

## Materials and Methods

### Materials

B27 and neurobasal media were obtained from Gibco (Grand Island, NY, USA). l-glutamine, fetal bovine serum (FBS), *N*-acetyl-Asp-Glu-Val-Asp-*p*-nitro-anilide (Ac-DEVD-*p*NA), dimethyl sulfoxide (DMSO), HEPES, CHAPS, mouse monoclonal anti-MAP2 antibody, ammonium persulfate, TEMED, TRIZMA base, Tween 20, dl-dithiothreitol, Nonidet NP-40, sodium deoxycholate, protease inhibitor (EDTA-free), bromophenol blue, 2′,7′-dichlorofluorescein diacetate, RIPA buffer, the Imprint Methylated DNA Quantification Kit, the Histone Deacetylase Assay Kit, the Histone Acetyltransferase Activity Assay Kit, the Autophagy Assay Kit, protease inhibitor cocktail for mammalian tissues, and poly-ornithine were obtained from Sigma-Aldrich (St. Louis, MO, USA). Bradford reagent, SDS, 30% acrylamide, 0.5 M Tris-HCl buffer, 1.5 M Tris-HCl gel buffer, and Laemmli sample buffer were from Bio-Rad Laboratories (Munchen, Germany). DHA and HX 531 were from Tocris Bioscience (Minneapolis, MN, USA). 2-Mercaptoethanol was from Carl Roth GmbH + Co. KG (Karlsruhe, Germany). Immobilon-P membranes were purchased from Millipore (Bedford, MA, USA). Alexa 488-conjugated anti-goat IgG, calcein AM, and Hoechst 33342 were purchased from Molecular Probes (Eugene, OR, USA). Cy3-conjugated anti-rabbit IgG and Cy5-conjugated anti-mouse were obtained from Jackson ImmunoResearch, Inc. (West Grove, PA, USA). The cytotoxicity detection kit and BM chemiluminescence western blotting substrate (POD) were purchased from Roche Diagnostics GmbH (Mannheim, Germany). ELISA kits for RXRα, RXRβ, RXRγ, LC3A, and LC3B were purchased from Shanghai Sunred Biological Technology Co. (Sunred, China). The culture dishes were obtained from TPP Techno Plastic Products AG (Trasadingen, Switzerland). The rabbit polyclonal anti-RXRα antibody (sc-774), mouse monoclonal anti-RXRβ antibody (sc-56869), mouse monoclonal anti-RXRγ antibody (sc-514134), mouse monoclonal anti-β-actin antibody (sc-47778), as well as RXRα siRNA (sc-36448), RXRβ siRNA (sc-36446) and RXRγ siRNA (sc-38879) were purchased from Santa Cruz Biotechnology, Inc. (Santa Cruz, CA, USA). AllStars Negative Control siRNA AF 488, the RNeasy Mini Kit, RT^2^ First Strand Kit, and RT^2^ Profiler PCR Autophagy Array were obtained from Qiagen (Valencia, CA, USA). INTERFERin was obtained from PolyPlus Transfection (Illkirch, France), and the High Capacity cDNA-Reverse Transcription Kit, the TaqMan Gene Expression Master Mix, and TaqMan probes for specific genes encoding hypoxanthine phosphoribosyltransferase coding gene (*Hprt*), *Rxrα*, *Rxrβ*, and *Rxrγ* were obtained from Life Technologies Applied Biosystems (Foster City, CA, USA). Quick-gDNA™ MicroPrep was obtained from Zymo Research (Irvine, CA, USA).

### Primary Neocortical Cell Cultures

Neocortical tissue for primary cultures was prepared from Swiss mouse embryos (Charles River, Germany) at 15–17 days of gestation and cultured as previously described [22, 29]. All procedures were performed in accordance with the National Institutes of Health Guidelines for the Care and Use of Laboratory Animals and were approved by the Bioethics Commission in compliance with Polish Law (21 August 1997). Animal care followed official governmental guidelines, and all efforts were made to minimize suffering as well as the number of animals used. The cells were suspended in estrogen-free neurobasal medium supplemented with B27 on poly-ornithine (0.01 mg/ml)-coated multi-well plates at a density of 2.0 × 10^5^ cells per cm^2^. The cultures were maintained at 37 °C in a humidified atmosphere containing 5% CO_2_ for 7 days in vitro (DIV) prior to experimentation. The number of astrocytes, as determined by the content of intermediate filament glial fibrillary acidic protein (GFAP), did not exceed 10% for all cultures [22, 30].

### Treatment

Primary neuronal cell cultures were exposed to BP-3 (10–100 μM) for 6 or 24 h. The involvement of RXR signaling in BP-3-induced effects was verified with the high-affinity RXR antagonist HX 531 (0.1 μM) and the RXR agonist DHA (1 μM) as previously described [22]. Specific ligands were added to the culture media 45–60 min before BP-3. To avoid nonspecific effects in our study, agonist and antagonist of RXRs were used at concentrations that did not affect the levels of caspase-3 activity or LDH release. All the compounds were originally dissolved in DMSO and were then further diluted in culture medium to maintain DMSO concentrations below 0.1%. The control cultures were supplemented with DMSO in concentrations that were equal to those used in the experimental groups.

### Identification of Apoptotic Cells

Apoptotic cells were detected via Hoechst 33342 staining at 24 h after the initial treatment as previously described [22, 29]. Neocortical cells that were cultured on glass coverslips were washed with 10 mM phosphate-buffered saline (PBS) and stained with Hoechst 33342 (0.6 mg/ml) at room temperature (RT) for 5 min. The cells containing bright blue fragmented nuclei, which was indicative of condensed chromatin, were identified as apoptotic cells. Qualitative analysis was performed using a fluorescence microscope (NIKON Eclipse 80i, NIKON Instruments Inc., Melville, New York, USA) equipped with a camera with the BCAM Viewer© Basler AG software. The level of cellular fluorescence from fluorescence microscopy images was determined using ImageJ software. To calculate the corrected total cell fluorescence (CTCF), the following equation was used: CTCF = Integrated density − (Area of selected cell × Mean fluorescence of background).

### Staining with Calcein AM

Intracellular esterase activity in the neocortical cultures was measured by calcein AM staining at 24 h after the initial treatment with BP-3 as previously described [22, 29]. To avoid the esterase activity present in the growth media, the cells were washed with PBS and incubated in 2 μM calcein AM in PBS at RT for 10 min. The cells displaying bright green cytoplasm were identified as live cells. Fluorescence intensity was monitored at Ex/Em 494/520 nm using a fluorescence microscope (NIKON Eclipse 80i, NIKON Instruments Inc., Melville, New York, USA) equipped with a camera with the BCAM Viewer© Basler AG software. The level of cellular fluorescence from fluorescence microscopy images was determined using ImageJ software. To calculate the CTCF, the following equation was used: CTCF = Integrated density − (Area of selected cell × Mean fluorescence of background).

### Assessment of Caspase-3 Activity

Caspase-3 activity was determined according to the protocol described by Nicholson (1995) using samples treated for 6 or 24 h with BP-3 alone or in combination with the test compounds. The assessment of caspase-3 activity was performed as previously described [22, 30, 31]. Cell lysates from neocortical cultures were incubated at 37 °C using Ac-DEVD-*p*NA, a colorimetric substrate that is preferentially cleaved by caspase-3. The levels of *p*-nitroanilide were continuously monitored for 60 min using a Multimode Microplate Reader Infinite M200PRO (Tecan, Mannedorf, Switzerland). The data were analyzed using the Magellan software, normalized to the absorbance of vehicle-treated cells, and expressed as a percentage of control ± SEM from three to four independent experiments. The absorbance of blanks, which acted as our enzyme-less control, was subtracted from each value.

### Measurement of Lactate Dehydrogenase Activity

To quantify cell death, lactate dehydrogenase (LDH) that was released from damaged cells into the cell culture media was measured 6 or 24 h after treatment with BP-3. LDH release was measured as previously described [22, 32]. Cell-free supernatants from neocortical cultures were collected from each well and incubated at room temperature for 30 to 60 min with the appropriate reagent mixture according to the manufacturer’s instructions (Cytotoxicity Detection Kit) depending on the reaction progress. The intensity of the red color that formed in the assay was measured at a wavelength of 490 nm (Infinite M200pro microplate reader, Tecan Mannedorf, Switzerland) and was proportional to both LDH activity as well as the number of damaged cells. The data were analyzed using the Magellan software, normalized to the color intensity from vehicle-treated cells (100%), and expressed as a percentage of the control value from three to four independent experiments. The absorbance of blanks, which acted as our enzyme-less control, was subtracted from each value.

### Silencing of RXRα, RXRβ, and RXRγ

Specific siRNAs were used to inhibit RXRα, RXRβ, and RXRγ expression in neocortical cells. Each siRNA was applied separately for 6 h at 50 nM in antibiotic-free medium containing the siRNA transfection reagent INTERFERin™ as previously described [22]. After transfection, the culture media were changed, and the cells were incubated for 12 h before starting the experiment. Positive and negative siRNAs containing a scrambled sequence that did not lead to the specific degradation of any known cellular mRNA were used as controls. The effectiveness of mRNA silencing was verified through the measurement of specific mRNAs using qPCR.

### qPCR Analysis of mRNAs Encoding the Receptors *Rxrα*, *Rxrβ*, and *Rxrγ*

Total RNA was extracted from neocortical cells that were cultured for 7 DIV (approx. 1.5 × 10^6^ cells per sample) using the RNeasy Mini Kit (Qiagen, Valencia, CA) according to the manufacturer’s instructions. The quantity of RNA was spectrophotometrically determined at 260 and 260/280 nm (ND/1000 UV/Vis; Thermo Fisher NanoDrop, USA). Two-step real-time quantitative polymerase chain reaction (qPCR) was performed as previously described [22]. Both the reverse transcription reaction and qPCR were run on a CFX96 Real-Time System (BioRad, USA). The products of the reverse transcription reaction were amplified using TaqMan Gene Expression Master Mix containing TaqMan primer probes specific to the genes encoding *Hprt*, *Rxrα*, *Rxrβ*, and *Rxrγ.* Amplification was performed in a total volume of 20 μl containing 10 μl of TaqMan Gene Expression Master Mix and 1.0 μl of reverse transcription product as the PCR template. A standard qPCR procedure was utilized: 2 min at 50 °C and 10 min at 95 °C followed by 40 cycles of 15 s at 95 °C and 1 min at 60 °C. The threshold value (Ct) for each sample was set during the exponential phase, and the delta Ct method was used for data analysis. *Hprt* was used as a reference gene.

### Mouse Autophagy RT^2^ Profiler PCR Array

Total RNA was extracted from neocortical cells cultured for 7 DIV (approx. 1.5 × 10^6^ cells per sample) using the RNeasy Mini Kit (Qiagen, Valencia, CA) according to the manufacturer’s instructions. The quantity of RNA was spectrophotometrically determined at 260 and 260/280 nm (ND/1000 UV/Vis; Thermo Fisher NanoDrop, USA). A total of 1 μg of mRNA was reverse-transcribed to cDNA using the RT^2^ First Strand Kit (Qiagen, Valencia, CA) and suspended in a final volume of 20 μl as previously described [13]. Each cDNA was prepared for further use in qPCR. To analyze the signaling pathway, the RT^2^ Profiler™ PCR Array System (Qiagen, Valencia, CA) was used according to the manufacturer’s protocol. The Ct values for all wells were exported to a blank Excel spreadsheet and were analyzed with the Web-based software (www.SABiosciences.com/pcrarraydataanalysis.php).

### Western Blot Analysis

The cells exposed to BP-3 for 24 h were lysed in ice-cold RIPA lysis buffer containing a protease inhibitor cocktail. The lysates were sonicated and centrifuged at 15,000×g for 20 min at 4 °C. The protein concentrations in the supernatants were determined using Bradford reagent (Bio-Rad Protein Assay) with bovine serum albumin (BSA) as the standard. Samples containing 40 μg of total protein were reconstituted in the appropriate amount of sample buffer comprised of 125 mM Tris, pH 6.8, 4% SDS, 25% glycerol, 4 mM EDTA, 20 mM DTT, and 0.01% bromophenol blue, denatured, and separated on a 7.5% SDS-polyacrylamide gel using a Bio-Rad Mini-Protean II Electrophoresis Cell as previously described [22, 33, 34]. After electrophoretic separation, the proteins were electrotransferred to PVDF membranes (Millipore, Bedford, MA, USA) using the Bio-Rad Mini Trans-Blot apparatus. Following the transfer, the membranes were washed, and the nonspecific binding sites were blocked with 5% dried milk and 0.2% Tween-20 in 0.02 M Tris-buffered saline (TBS) for 2 h with shaking. The membranes were incubated overnight (at 4 °C) with one of the following primary antibodies (Santa Cruz Biotechnology) diluted in TBS/Tween: anti-RXRα rabbit polyclonal antibody (diluted 1:150), anti-RXRβ mouse monoclonal antibody (diluted 1:100), anti-RXRγ mouse monoclonal antibody (diluted 1:100), or anti-β-actin mouse monoclonal antibody (diluted 1:3000). The signals were developed by chemiluminescence (ECL) using BM Chemiluminescence Blotting Substrate (Roche Diagnostics GmBH) and visualized using a Luminescent Image Analyzer Fuji-Las 4000 (Fuji, Japan). Immunoreactive bands were quantified using a MultiGauge V3.0 image analyzer.

### Enzyme-Linked Immunosorbent Assays for RXRα, RXRβ, and RXRγ

The levels of RXRα, RXRβ, RXRγ, LC3A, and LC3B were determined in neocortical cells 24 h after treatment with BP-3 as previously described [22]. Specific detection of these proteins was achieved using ELISAs and the quantitative sandwich enzyme immunoassay technique. A 96-well plate was precoated with monoclonal antibodies that were specific for RXRα, RXRβ, RXRγ, LC3A, and LC3B. The standards and non-denatured cell extracts were added to the wells with biotin-conjugated polyclonal antibodies specific for RXRα, RXRβ, RXRγ, LC3A, and LC3B. Therefore, all native RXRα, RXRβ, RXRγ, LC3A, and LC3B proteins were captured using the immobilized antibodies. The plates were washed to remove any unbound substances, and horseradish peroxidase-conjugated avidin was added to interact with the biotin bound to RXRα, RXRβ, RXRγ, LC3A, and LC3B. After washing, the substrate solution was added to the wells. The enzymatic reaction yielded a blue product. The absorbance was measured at 450 nm and was proportional to the amount of RXRα, RXRβ, RXRγ, LC3A, and LC3B in the sample. The protein concentration was determined in each sample using Bradford reagent—Bio-Rad Protein Assay [22, 35].

### Immunofluorescence Labeling of RXRα, RXRβ, and RXRγ and Confocal Microscopy

For immunofluorescence detection of RXRα, RXRβ, and RXRγ, neocortical cells were cultured on glass coverslips and subjected to immunofluorescence double-labeling as previously described [22, 36]. After 1 h of incubation in a blocking buffer (5% normal donkey serum and 0.3% Triton X-100 in 0.01 M PBS), the cells were treated for 24 h (at 4 °C) using four primary antibodies: rabbit polyclonal anti-RXRα antibody (1:50), mouse monoclonal anti-RXRβ antibody (1:50), mouse monoclonal anti-RXRγ antibody (1:50), and anti-MAP2 mouse monoclonal antibody (1:100) followed by a 24-h incubation in a mixture of secondary antibodies, including Cy3-conjugated anti-rabbit IgG (1:300) and Cy5-conjugated anti-mouse IgG (1:300). The samples were subsequently washed, mounted, coverslipped, and analyzed using an LSM510 META, Axiovert 200M confocal laser scanning microscope (Carl Zeiss MicroImaging GmbH, Jena, Germany) under a Plan-Neofluor 40×/1.3 Oil DIC objective. A He/Ne laser and an argon laser, with two laser lines emitting at 514 and 633 nm, were used to excite the Cy3-, and Cy5-conjugated antibodies, respectively. The fluorescence signal was enhanced after combining four scans per line. A pinhole value of 1 airy unit was used to obtain flat images.

### Measurement of Global DNA Methylation

Genomic DNA was extracted from neocortical tissues using the Quick-gDNA™ MicroPrep kit (Zymo Research, Irvine, CA) according to the manufacturer’s instructions. The quantity of DNA was spectrophotometrically determined at 260 and 260/280 nm (ND/1000 UV/Vis; Thermo Fisher NanoDrop, USA). Global DNA methylation changes were measured in neocortical cells at 24 h after treatment using a specific ELISA-based kit (Imprint® Methylated DNA Quantification—Sigma-Aldrich; St. Louis, MO, USA) as previously described [22]. This kit contained all the reagents required to determine the relative levels of methylated DNA. The methylated DNA was detected using the capture and detection antibodies and quantified colorimetrically using an Infinite M200pro microplate reader (Tecan, Austria). The amount of methylated DNA present in the sample was proportional to the absorbance measured.

### Detection of Autophagosomes

Cultured cells on 96-well plates were treated according to the manufacturer’s instructions. The Autophagy Assay kit provided a simple and direct procedure for measuring autophagy in a variety of cell types using a proprietary fluorescent autophagosome marker (*λ*_ex_ = 333/*λ*_em_ = 518 nm). The autophagosomes were detected using an Infinite M200pro microplate reader (Tecan, Austria).

### Measurement of HDAC and HAT Activity

The HDAC and HAT activities were detected using the Histone Deacetylase Assay Kit and the Histone Acetyltransferase Activity Fluorometric Assay Kit (Sigma-Aldrich, St. Louis, MO, USA) according to the manufacturer’s instructions. Regarding HDAC kit, the measured fluorescence at *λ*_ex_ = 365 nm/*λ*_em_ = 460 nm was proportional to the deacetylation activity. In the HAT assay, the generated product of histone acetyltransferase activity was detected fluorimetrically at *λ*_ex_ = 535/*λ*_em_ = 587 nm. The kit included an active nuclear extract to be used as a positive control. The abovementioned assays provided positive and negative controls.

### Data Analysis

Statistical tests were performed on raw data that were expressed as the mean arbitrary absorbance or as the fluorescence units per well containing 50,000 cells (measurements of caspase-3, LDH, autophagosomes; the fluorescence units per 1.5 million cells (qPCR, global DNA methylation, and HDAC and HAT activity); the mean optical density per 40 μg of protein (western blotting); or picograms of RXRα, RXRβ, RXRγ, LC3A, and LC3B per micrograms of total protein (ELISA). Statistical analysis of cellular fluorescence related to Hoechst 33342 and calcein AM staining was performed on CTCF data using 40 counts per image. One-way analysis of variance (ANOVA) was preceded by the Levene’s test of homogeneity of variances and was used to determine overall significance. Differences between the control and experimental groups were assessed using a post hoc Newman–Keuls test, and significant differences were designated as ^*^ *p* < 0.05, ^**^ *p* < 0.01, and ^***^ *p* < 0.001 versus control cultures; ^#^ *p* < 0.05, ^##^ *p* < 0.01, and ^###^ *p* < 0.001 versus the cultures exposed to BP-3; and ^$^ *p* < 0.05 and ^$$$^ *p* < 0.001 versus the siRNA-transfected control cultures. The results were expressed as the mean ± SEM of three to four independent experiments. The number of replicates in each experiment ranged from 2 to 3, except for the measurements of caspase-3 activity and LDH release, which contained five to eight replicates. To compare the effects of BP-3 in different treatment paradigms, the results for the caspase-3, LDH, ELISA, and western blot analyses were presented as a percentage of the control.

## Results

### Effects of BP-3 on Caspase-3 Activity and LDH Release in Neocortical Cultures at 7 DIV

**Fig. 1:**
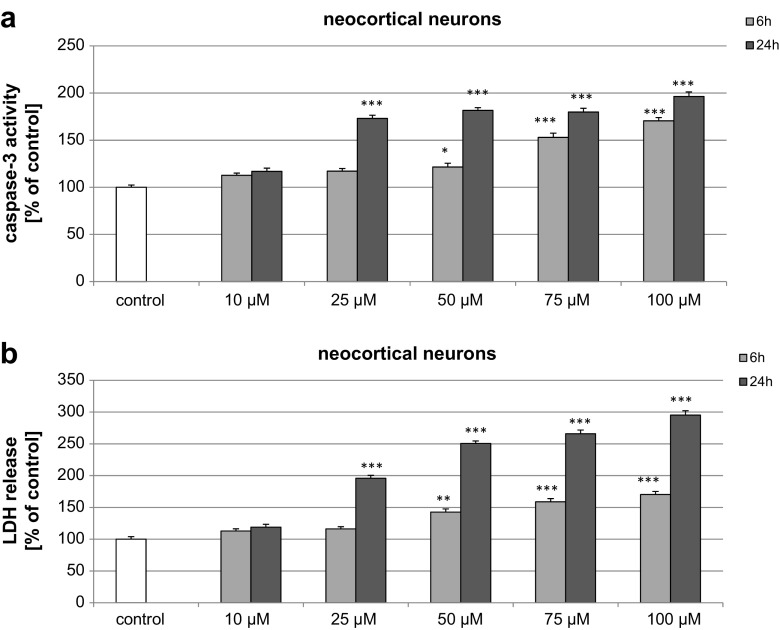
Time-course effects of BP-3 (10, 25, 50, 75, and 100 μM) on caspase-3 activity (**a**) and LDH release (**b**) in primary cultures of mouse neocortical cells at 7 DIV. The cells were treated with BP-3 for 6 and 24 h. The results are presented as a percentage of the control. Each *bar* represents the mean of three to four independent experiments ± SEM. The number of replicates in each experiment ranged from 5 to 8. ^*^ *p* < 0.05, ^**^ *p* < 0.01, and ^***^ *p* < 0.001 versus control cultures

In neocortical cultures at 7 DIV, BP-3 (25–100 μM) induced an increase in caspase-3 levels to 170% of the control level at 6 h, which were further enhanced to 196% at 24-h post-treatment (Fig. 1a). In these cells, LDH release from neocortical cells increased in a time-dependent manner to 150–180% of the control value at 6 h and to 200–290% at 24 h (Fig. 1b).

### Effects of BP-3 Alone or in Combination with HX 531 on Hoechst 33342 and Calcein AM Staining in Neocortical Cultures

**Fig. 2:**
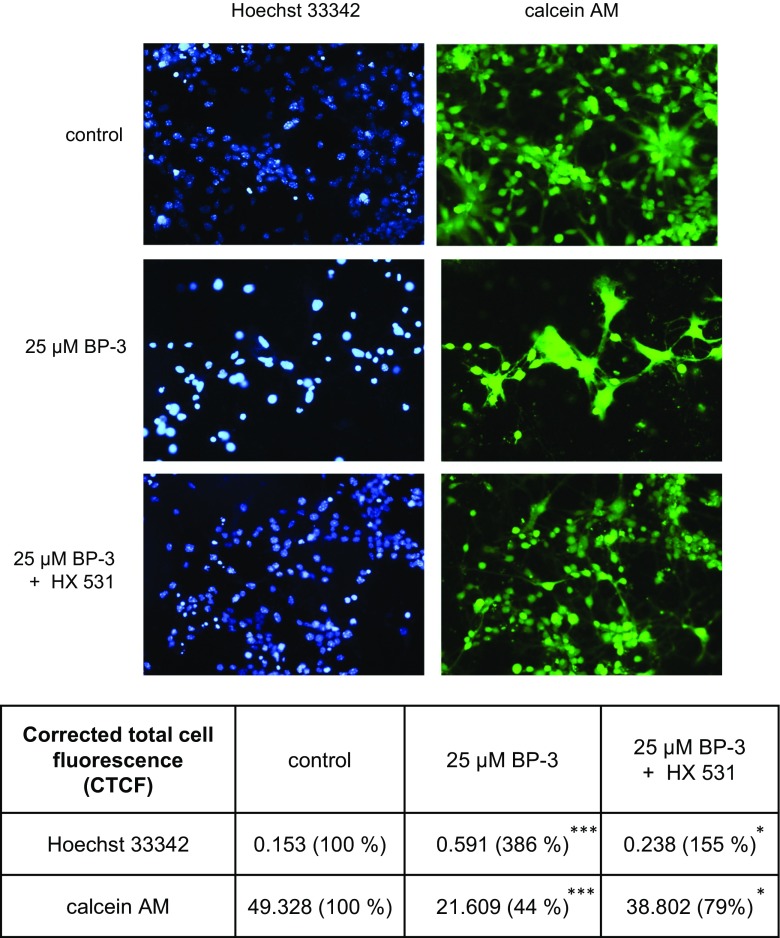
Influence of BP-3 (25 μM) and HX 531 (0.1 μM) on Hoechst 33342 (*first column*) and calcein AM (*second column*) staining in mouse neocortical cultures at 7 DIV, examined 24 h post-treatment. Cells with bright fragmented nuclei with condensed chromatin were identified as cells undergoing apoptosis, whereas cells with light-colored cytoplasm were identified as live cells. Statistical analysis of relevant fluorescence signals was performed on CTCF data using 40 counts per image. ^*^ *p* < 0.05 and ^***^ *p* < 0.001 versus control cultures

In the present study, a 24-h exposure to BP-3 (25 μM) was necessary to develop an apoptotic morphology in cell nuclei. Apoptotic cells were detected by Hoechst 33342 staining as formation of bright blue fragmented nuclei containing condensed chromatin (Fig. 2). Furthermore, treatment with BP-3 reduced the density of calcein AM-stained living cells at 7 DIV, as indicated by the decreased number of cells exhibiting light-colored cytoplasm. Co-treatment with RXR antagonist-HX 531 (0.1 μM) inhibited the BP-3-induced effects. Quantitative analysis of relevant fluorescence signals showed that at 7 DIV, 25 μM BP-3 caused an increase in formation of condensed chromatin by 386% of the control level and reduced number of live cells by 56%. Treatment with HX 531 (0.1 μM) inhibited the effect of BP-3 in respect to the apoptotic fragmentation of cell nuclei by 231% and enhanced cell viability by 35% (Fig. 2).

### Effects of BP-3 Alone or in Combination with DHA and HX 531 on BP-3-Induced Caspase-3 Activity and LDH Release in Neocortical Cultures

**Fig. 3:**
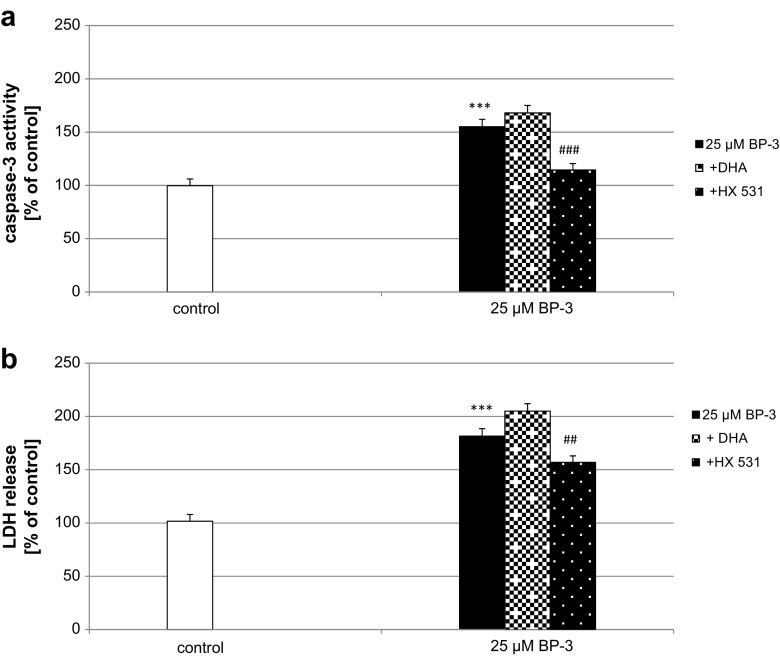
Impact of the RXR agonist and antagonist on BP-3-induced caspase-3 activity (**a**) and LDH release (**b**) in neocortical cultures at 7 DIV. The primary neocortical cultures were treated with BP-3 (25 μM) for 24 h. The results were normalized to the absorbance of vehicle-treated cells and are expressed as a percentage of control. Each *bar* represents the mean of three to four independent experiments ± SEM. The number of replicates in each experiment ranged from 5 to 8. ^***^ *p* < 0.001 versus control cultures; ^##^ *p* < 0.01 and ^###^ *p* < 0.001 versus the cultures exposed to BP-3

Neocortical cultures exposed to BP-3 (25 μM) for 24-h caused a greater than 50% increase in caspase-3 activity in the neuronal cells. Co-treatment with the selective RXR agonist DHA (1 μM) did not change the effect of BP-3 on caspase-3 activity. The RXR antagonist HX 531 (0.1 μM) inhibited the BP-3 (25 μM)-induced caspase-3 activity by 40% (Fig. 3a).

The selective RXR agonist DHA (1 μM) did not affect BP-3-induced LDH release. However, the high-affinity RXR antagonist HX 531 (0.1 μM) diminished BP-3-induced LDH release by 25% (Fig. 3b).

### Effect of BP-3 on the mRNA Levels of *Rxrα*, *Rxrβ*, and *Rxrγ*

**Fig. 4:**
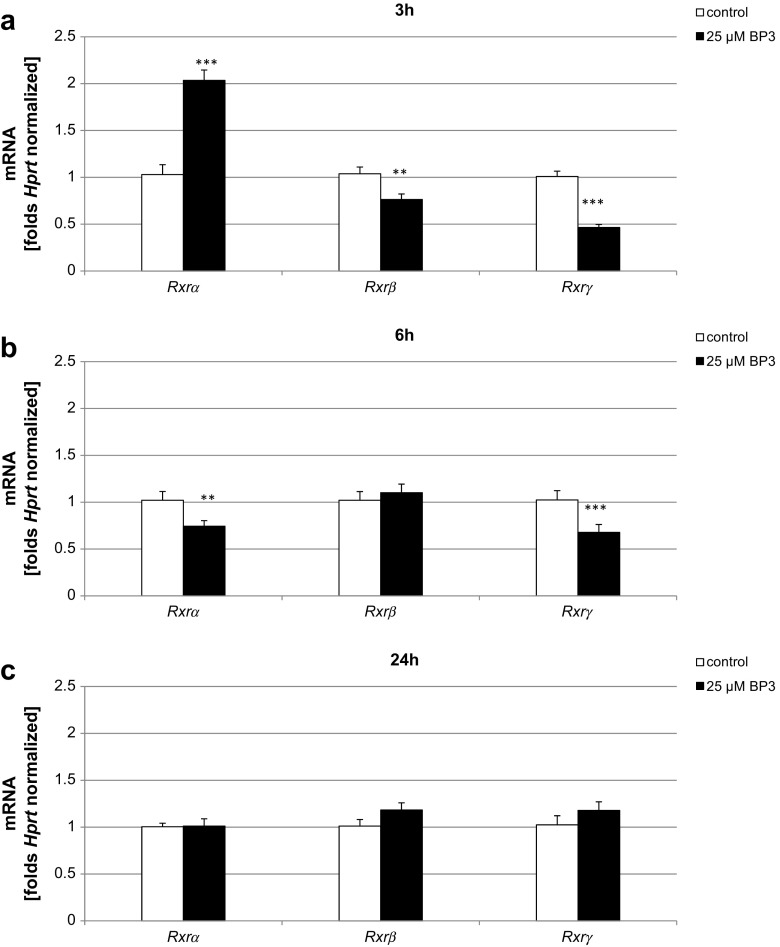
Effect of BP-3 (25 μM) on the mRNA expression levels of *Rxrα*, *Rxrβ*, and *Rxrγ* in neocortical cultures at 7 DIV. Each *bar* represents the mean ± SEM of three independent experiments. The number of replicates for each experiment ranged from 2 to 3. ^**^ *p* < 0.01 and ^***^ *p* < 0.001 versus control cultures

According to our data, treatment with BP-3 (25 μM) affected the mRNA levels of *Rxrα*, *Rxrβ*, and *Rxrγ*. A 3-h exposure of the neocortical cultures to BP-3 caused a 25% decrease in *Rxrβ* and a 55% decrease in *Rxrγ* but caused a 100% increase in *Rxrα* mRNA compared with the control (Fig. 4a). The pattern of mRNA expression was changed after prolonged exposure to BP-3. After 6 h of treatment, BP-3 decreased the mRNA expression level of *Rxrα* (26%) but did not change the expression level of *Rxrβ* in neocortical cells (Fig. 4b). After 24 h of exposure, BP-3 did not change the mRNA expression levels of any *Rxrs* (Fig. 4c). These data were normalized to *Hprt*.

### Effects of BP-3 on the Protein Expression Levels of RXRα, RXRβ, and RXRγ in Mouse Neocortical Cells

**Fig. 5:**
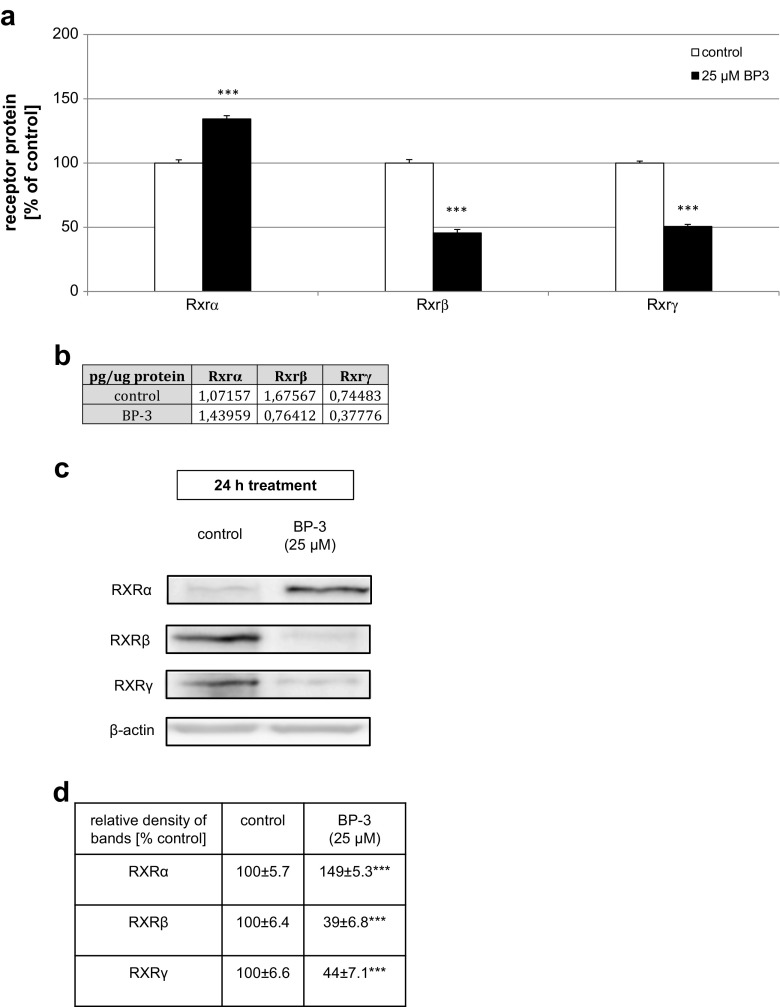
Effects of BP-3 on the protein levels of RXRα, RXRβ, and RXRγ in mouse neocortical cultures at 7 DIV. The neocortical cells were cultured for 7 DIV and then treated for 24 h with BP-3 (25 μM). The concentrations of the receptors were measured using specific ELISAs and are presented as a percentage of the control (**a**) and as picogram of specific protein, i.e., RXRα, RXRβ, and RXRγ, per microgram of total protein (**b**). For the western blot analyses, protein samples were denatured, electrophoretically separated, transferred to PVDF membranes, and subjected to immunolabeling (**c**). The relative protein levels of RXRα, RXRβ, and RXRγ were presented as a percentage of the control (**d**). Each *bar* or value represents the mean of three independent experiments ± SEM. The number of replicates in each experiment ranged from 2 to 3. ^***^ *p* < 0.001 versus control cultures

A 24-h exposure to BP-3 was necessary to detect changes in the protein levels of the receptors. In the cultures treated with BP-3, RXRα expression enhanced and reached 1.43 pg/μg of total protein (134% of the control). In cultures exposed to BP-3 (25 μM) for 24 h, the concentration of RXRβ was 0.76 pg/μg of total protein, and it was 56% less than that in control cultures. In the cultures exposed to BP-3, the level of RXRγ reached 0.38 pg/μg, which was 49% less than controls (Fig. 5a, b).

Western blot analysis determined the relative protein expression levels of RXRα, RXRβ, and RXRγ in mouse neocortical cells at 7 DIV. Exposure to BP-3 (25 μM) for 24 h decreased the relative RXRβ and RXRγ protein levels by 61 and 56%, respectively. Treatment with BP-3 (25 μM) increased the relative RXRα protein level by 49% (Fig. 5c, d).

### Effect of BP-3 on the Distribution of RXRα, RXRβ, RXRγ, and MAP2 Staining in Neocortical Cells

**Fig. 6:**
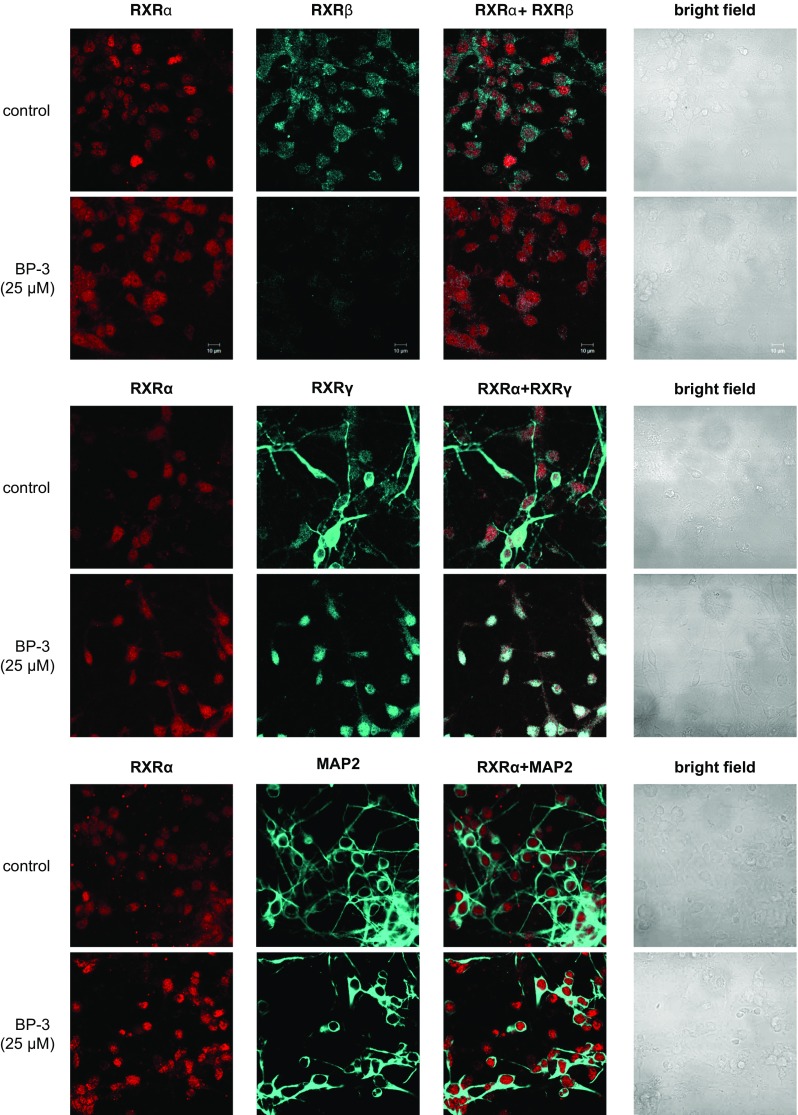
Influence of BP-3 on the cellular distributions of RXRα (*red*), RXRβ (*blue*), RXRγ (*blue*), and MAP2 (*blue*) in mouse neocortical cultures at 7 DIV. The overlay of RXRα/RXRβ, RXRα/RXRγ, and RXRα/MAP2 (*red plus blue*) staining with the bright field images are shown. The primary neocortical cultures were treated with BP-3 (25 μM) for 24 h. Analyzed using an LSM510 META, Axiovert 200M confocal laser scanning microscope under a Plan-Neofluor 40×/1.3 Oil DIC objective

Immunofluorescence labeling was performed in parallel with the measurements of receptor protein levels. Confocal microscopy revealed that RXRα, RXRβ, and RXRγ were localized to neocortical cells at 7 DIV. A 24-h exposure to BP-3 (25 μM) increased RXRα staining but reduced RXRβ- and RXRγ-specific immunofluorescence. MAP2 staining confirmed the neural localization of the receptors and revealed the BP-3-induced inhibition of neurite outgrowth (Fig. 6).

### Influence of BP-3 on Caspase-3 Activity and LDH Release in Neocortical Cells Transfected with RXRα, RXRβ, and RXRγ siRNAs

**Fig. 7:**
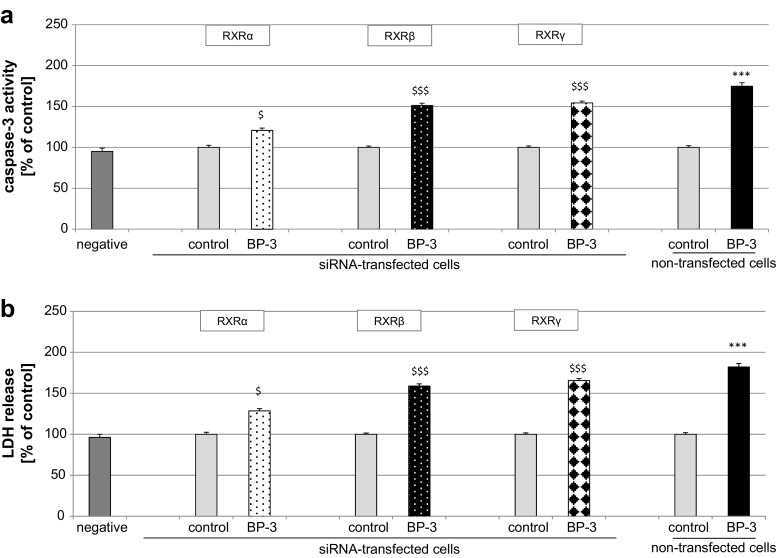
Effect of BP-3 (25 μM) on caspase-3 activity (**a**) and LDH release (**b**) in RXRα, RXRβ, and RXRγ siRNA-transfected neocortical cells. Each *bar* represents the mean ± SEM of three to four independent experiments. The number of replicates in each experiment ranged from 5 to 8. ^***^ *p* < 0.001 versus the non-transfected control cultures; ^$^ *p* < 0.05 and ^$$$^ *p* < 0.001 versus the siRNA-transfected control cultures

A 24-h exposure to BP-3 (25 μM) only slightly reduced caspase-3 activity and LDH release in the RXRβ and RXRγ siRNA-transfected cells, suggesting that the transfected cells were slightly less vulnerable to BP-3 than the non-transfected cells. In comparison to the non-transfected cells, the effects of BP-3 were reduced by 20% with respect to caspase-3 levels and by 30% with respect to LDH release in the siRNA-transfected cells (Fig. 7a, b).

A 24-h exposure of RXRα siRNA-transfected cells to 25 μM BP-3 reduced caspase-3 activity and LDH release to 55 and 50% of the control values, respectively (Fig. 7a, b). These cells were much less vulnerable to BP-3 than the non-siRNA-treated wild-type cells.

The effectiveness of mRNA silencing was verified by qPCR. In this study, siRNA treatment decreased the *Rxrα* mRNA level by 83% (equal to 0.17-fold), the *Rxrβ* mRNA level by 64% (equal to 0.36-fold), and the *Rxrγ* mRNA level by 68% (equal to 0.32-fold) compared to the non-transfected wild-type cells.

### Influence of BP-3 on Global DNA Methylation in Neocortical Cultures

**Fig. 8:**
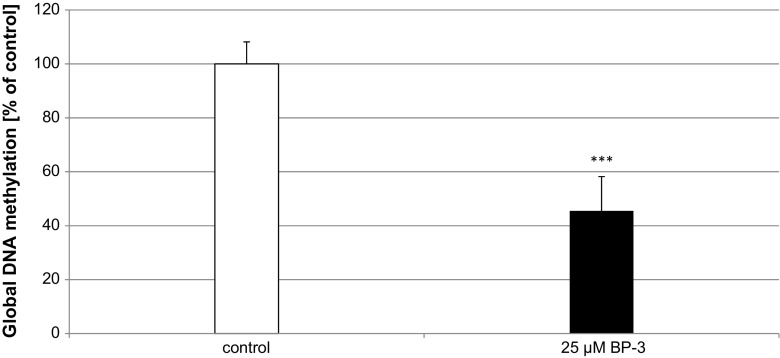
Influence of BP-3 on global DNA methylation in neocortical cultures at 7 DIV. Primary neocortical cultures were treated with BP-3 (25 μM) for 24 h. Total DNA was extracted from cells followed by ELISA. Each *bar* represents the mean of three independent experiments ± SEM. The number of replicates in each experiment ranged from 2 to 3. ^***^ *p* < 0.001 versus control cultures

A 24-h exposure of neocortical cells to BP-3 (25 μM) caused changes in the level of global DNA methylation. The treatment with BP-3 reduced the methylation level by 55% of the control value (Fig. 8).

### Effects of BP-3 on HDAC and HAT Activity in Neocortical Cultures

**Fig. 9:**
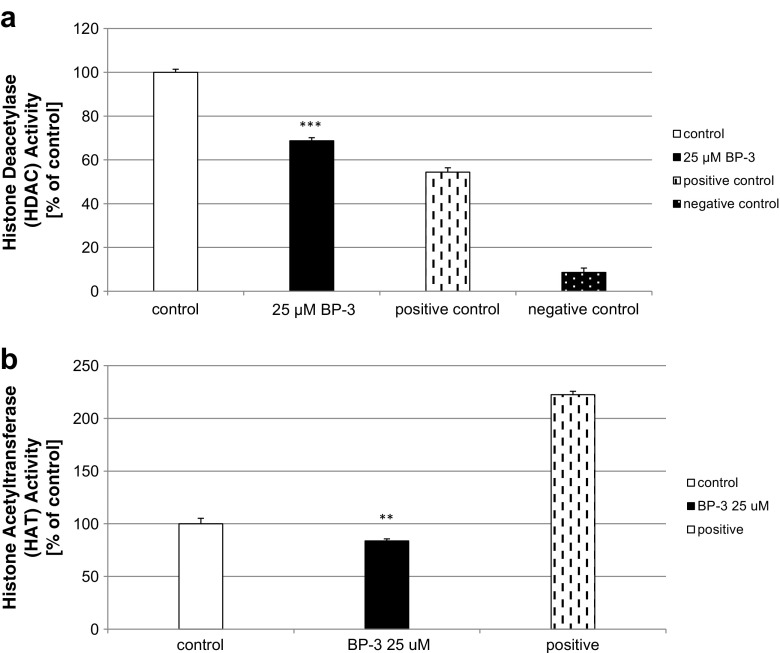
Effects of BP-3 (25 μM) on HDAC (**a**) and HAT (**b**) activity in the primary cultures of mouse neocortical cells at 7 DIV. The cells were treated with BP-3 for 24 h. The results are presented as a percentage of the control. Each *bar* represents the mean of three to four independent experiments ± SEM. The number of replicates in each experiment ranged from 5 to 8. ^**^ *p* < 0.01 and ^***^ *p* < 0.001 versus control cultures

A 24-h exposure of neocortical cultures to BP-3 reduced the levels of HDAC and HAT activities. Treatment with BP-3 decreased the activities of HDAC and HAT by 32 and 17% of the control value, respectively (Fig. 9a, b).

### Effects of BP-3 on the Expression Profiles of Genes Involved in Autophagy Using a Mouse Autophagy RT^2^ Profiler PCR Array

**Fig. 10:**
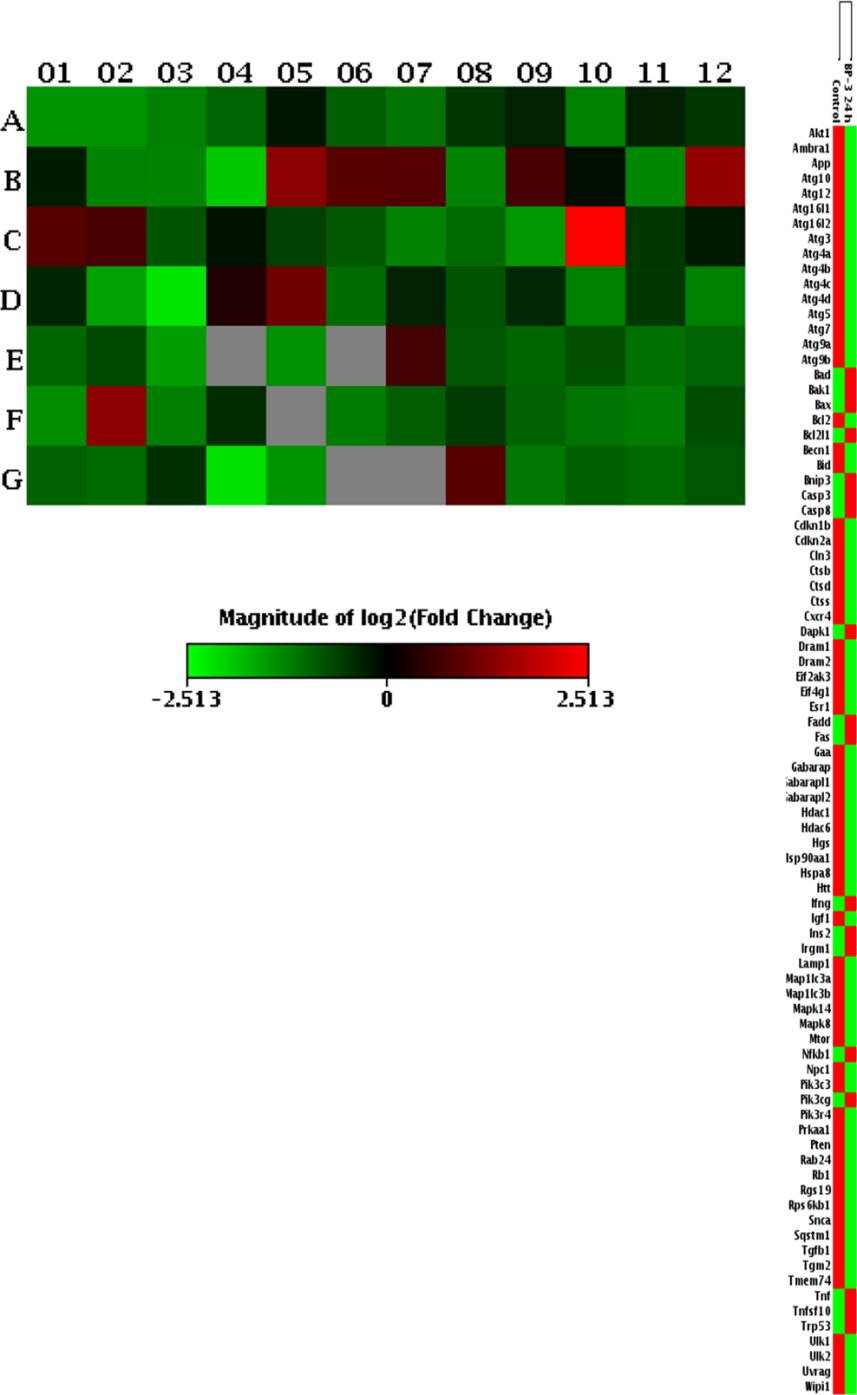
Gene expression patterns of autophagy showing the 71 genes that were significantly differentially expressed between the control and BP-3-treated groups. Among these genes, 60 genes were downregulated (*green color*) and 11 genes were upregulated (*red color*) in the BP-3-treated samples compared to the control

To validate that BP-3 impairs autophagy in neuronal cells, we analyzed a total number of 84 key genes that are involved in this process. Among them, 71 genes were differentially expressed in response to BP-3 treatment: 60 were downregulated (green color) and 11 were upregulated (red color) in the BP-3-treated samples. The downregulated genes were *Akt1*, *Ambra1*, *App*, *Atg10*, *Atg16l1*, *Atg16l2*, *Atg3*, *Atg4b*, *Atg4d*, *Atg7*, *Atg9a*, *Atg9b*, *Bcl2*, *Bid*, *Cdkn1b*, *Cdkn2a*, *Cln3*, *Ctsb*, *Ctsd*, *Ctss*, *Cxcr4*, *Dram1*, *Eif2ak3*, *Eif4g1*, *Esr1*, *Gaa*, *Gabarapl1*, *Gabarapl2*, *Hdac1*, *Hdac6*, *Hgs*, *Hsp90aa1*, *Hspa8*, *Htt*, *Igf1*, *Irgm1*, *Lamp1*, *Map1lc3a*, *Map1lc3b*, *Mapk14*, *Mapk8*, *Mtor*, *Npc1*, *Pik3c3*, *Pik3r4*, *Prkaa1*, *Pten*, *Rab24*, *Rb1*, *Rgs19*, *Rps6kb1*, *Snca*, *Sqstm1*, *Tgfb1*, *Tgm2*, *Tmem74*, *Ulk1*, *Ulk2*, *Uvrag*, and *Wipi1*. The upregulated genes were *Bad*, *Bak1*, *Bax*, *Bcl2l1*, *Bnip3*, *Casp3*, *Casp8*, *Dapk1*, *Fas*, *Nfkb1*, and *Trp53* (Fig. 10).

### Effects of BP-3 on Autophagosome Detection

**Fig. 11:**
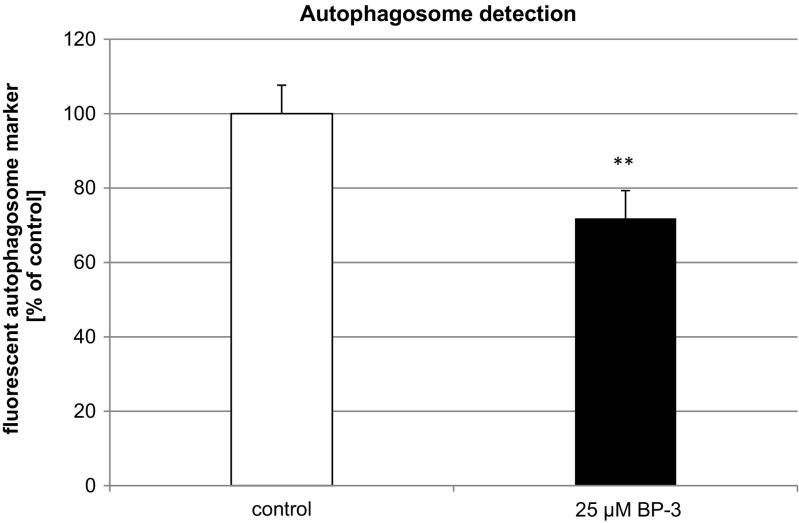
The effect of 25 μM BP-3 on autophagosome levels at 24 h. The data are expressed as the mean ± SEM of four independent experiments, consisting of eight replicates per treatment group. ^**^ *p* < 0.001 versus the control

A 24-h exposure of neocortical cultures to BP-3 reduced the level of autophagosomes in mouse neuronal cell cultures. Treatment with BP-3 decreased autophagosome level by 29% compared to the control value (Fig. 11).

### Effects of BP-3 on the Protein Expression Levels of LC3A and LC3B in Mouse Neocortical Cells

**Fig. 12:**
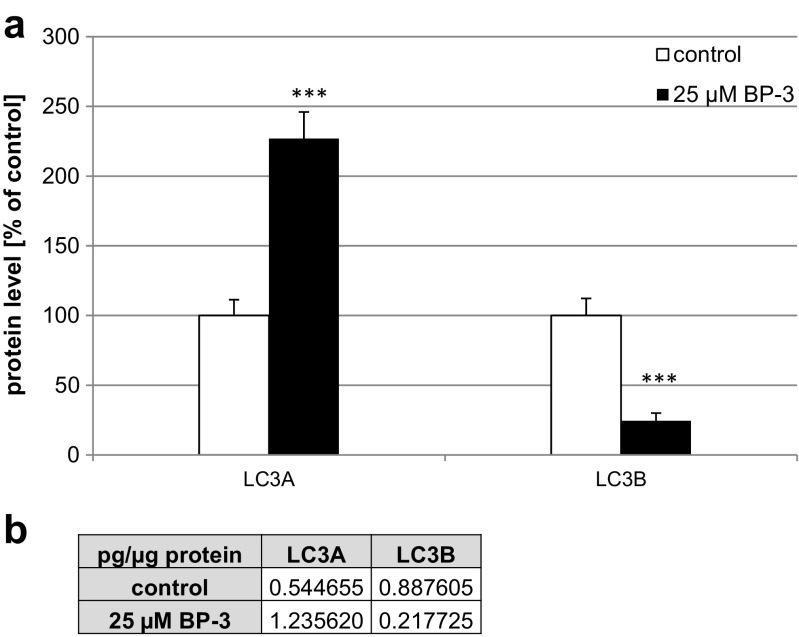
Effects of BP-3 on the protein levels of LC3A and LC3B in mouse neocortical cultures at 7 DIV. The neocortical cells were cultured for 7 DIV and then treated for 24 h with BP-3 (25 μM). The concentrations of the receptors were measured using ELISA and are presented as a percentage of the control (**a**) and as picogram of the specific protein (LC3A or LC3B) per microgram of total protein (**b**). ***p < 0.001 versus control cultures

In cultures exposed to BP-3 (25 μM) for 24 h, the concentration of LC3A was 1.23 pg/μg of total protein, which was 126% higher than that in control cultures. In the cultures exposed to BP-3, the level of LC3B reached 0.21 pg/μg, which was reduced by 75% compared to controls (Fig. 12a, b).

## Discussion

The primary aim of the present study was to evaluate the neurotoxic effects of BP-3 with an emphasis on apoptosis, autophagy, the epigenetic status of neuronal cells, and the molecular mechanisms involving RXRs. The results of the study demonstrated that BP-3 caused neurotoxicity, as evidenced by the concentration-dependent activation of caspase-3 and LDH release in mouse neocortical cells. In the present study, neocortical cells responded to 25–100 μM BP-3. This was in line with our previous study in which 25 μM BP-3 was determined to be the lowest effective concentration at 24 h of exposure [13]. The used concentration is environmentally relevant since BP-3 has been found in human adipose tissue at concentrations up to 5 mg/kg (~22 μM) [37]. The ability of BP-3 to cross the blood-brain barrier has been shown; after it was applied by gavage, the *Erα* and *Erβ* mRNA expression levels were changed in the rat pituitary gland [38]. Moreover, a BP-3 analogue (BP-4) changed the expression levels of many genes in the *Danio rerio* brain when it was added to water [39]. In the present study, the neurotoxic effects of BP-3 involved enhanced LDH release and impaired cell survival (calcein AM staining), which were accompanied by the induction of apoptosis as estimated by caspase-3 activation and increased apoptotic body formation (Hoechst 33342 staining).

In this study, both the neurotoxic and apoptotic effects of BP-3 were inhibited by HX 531, which is a potent RXR antagonist. These data suggest that RXR receptors are involved in BP-3-induced effects in neuronal cells. Previously, we demonstrated an important role of RXRα- and RXRβ-intracellular signaling in the propagation of DDE- and nonylphenol-induced apoptosis during the early stages of neural development [22–24]. Recently, the involvement of RXRs in apoptotic signaling in retina pigment epithelial cells and gastrointestinal cancer cells has been shown [40, 41]. The role of RXRs in neuronal survival and neurotoxicity is complex and depends on RXR heterodimerization partners. Interestingly, A/B domain region of RXR receptors was found to cause growth inhibition or rexinoid-induced apoptosis [42]. Elevated levels of RXRα gene and protein expression have been found in individuals suffering from dementia [43], and knockout of RXRγ impairs the working memory in mice [44]. In the present study, BP-3 altered the mRNA expression levels of *Rxrα*, *Rxrβ*, and *Rxrγ* in a time-dependent manner. Increased expression of *Rxrα* mRNA and reduced expression levels of *Rxrβ* and *Rxrγ* mRNA were observed at 3 h of exposure, which mirrored the alterations in the estimated protein levels of the receptors at 24 h of exposure. These profiles were also concurred with the immunofluorescent labeling of RXRα, RXRβ, and RXRγ following BP-3 treatment. Based on these data, we hypothesize that the BP-3-induced apoptosis of neuronal cells is mediated by the attenuation of RXRβ/RXRγ and the stimulation of RXRα signaling pathways. To test this hypothesis, we used gene-specific siRNAs. Compared to non-transfected wild-type cells, silencing of RXRα caused a substantial reduction of BP-3-induced caspase-3 activity and LDH release; however, silencing of RXRβ and RXRγ did not affect the BP-3-induced effects. Therefore, we suggest that the BP-3-induced apoptosis of neuronal cells is mediated by the stimulation of RXRα signaling and the attenuation of RXRβ/RXRγ signaling, which is in line with the BP-3-induced alterations in RXR expression levels.

Previously, we demonstrated that stimulation of ERβ/GPR30 and impairment of ERα/PPARγ signaling were involved in propagation of BP-3-induced apoptosis [13]. Taking into account our previous and present data, one may assume that in neuronal cells BP-3 stimulates RXRα/ERβ/GPR30 and inhibits RXRβ/RXRγ/ERα/PPARγ intracellular pathways. It has been documented that ERβ may downregulate ERα/PPARγ and disrupt RXRα/PPARγ signaling [45]. RXRα is an obligatory heterodimer partner of PPARγ as well as RXRβ/RXRγ [46]. We suggest that in our study, BP-3 by upregulation of ERβ impaired RXRβ/RXRγ/ERα/PPARγ signaling. We also postulate that BP-3 was able to destroy RXRβ and RXRγ heterodimers but not RXRα homodimers, possibly due to stronger covalent bonding in homodimers than in heterodimers.

In addition to the demonstration that the BP-3-induced apoptosis involves the activation of RXRα signaling and the impairment of RXRβ/RXRγ signaling, we showed that BP-3 inhibited global DNA methylation as well as reduced HDAC and HAT activities in mouse embryonic neuronal cells. Aberrant DNA methylation and other epigenetic modifications have been found to be associated with development and normal cellular homeostasis as well as growing number of human diseases. Low doses of a pesticide DDT have been postulated to cause hypomethylation of specific gene regions in the young brain and impaired hippocampal neurogenesis [25]. Recently, we showed evidence of the involvement of global DNA hypomethylation in DDT-induced depressive-like effects and the DDE-induced apoptosis of primary neuronal cells [22, 47]. Exposures to xenobiotics such as tributyltin (TBT) and triphenyltin (TPT) have been shown to alter HDAC and HAT activity [48]. The global DNA hypomethylation as well as diminished HDAC and HAT activity suggest of chromosomal instability thus can cause inappropriate gene expression pattern. We postulate that global DNA hypomethylation and diminished HDAC activity are responsible for the BP-3-induced increase in the RXRα expression level, whereas diminished HAT activity corresponds to reduced expression of RXRβ/RXRγ in response to BP-3 treatment in our study.

Based upon our data, we suggest that altered epigenetic status caused by BP-3 treatment may not only be involved in apoptosis and the disruption of RXR signaling but may also affect autophagy. Autophagy is neuroprotective and is responsible for degrading damaged organelles and misfolded proteins. Dysregulation of autophagy has been linked to neural degenerative diseases such as Alzheimer’s disease, Parkinson’s disease, Huntington’s disease, amyotrophic lateral sclerosis, and encephalopathy. In the present study, the attenuation of the autophagic process was confirmed by the downregulation of genes involved in autophagy as detected by the microarray analysis, decreased autophagosome formation, and the reduced ratio of LC3B to LC3A. We hypothesize that BP-3 induced the downregulation of genes related to autophagy through decreased HAT activity in mouse neurons.

## Conclusions

In summary, we showed for the first time that the BP-3-induced apoptosis of neuronal cells is mediated via the stimulation of RXRα signaling and the attenuation of RXRβ/RXRγ signaling, as demonstrated by the use of selective antagonist and specific siRNAs as well as by measuring the mRNA and protein expression levels (qPCR, ELISA, western blot, and immunofluorescent labeling) of the receptors. This study also demonstrated that the use of BP-3 at environmentally relevant concentrations was able to inhibit autophagy and disrupt the epigenetic status of neuronal cells, which may increase the risk of neurodevelopmental abnormalities and/or neural degeneration.

## Abbreviations

AM: acetoxymethyl
ANOVA: analysis of variance
BP-3: benzophenone-3
BPA: bisphenol A
BPs: benzophenones
DDE: dichlorodiphenyldichloroethylene
DIV: days in vitro
DMSO: dimethyl sulfoxide
EDCs: endocrine disrupting chemicals
ELISA: enzyme-linked immunosorbent assay
FBS: fetal bovine serum
GFAP: glial fibrillary acidic protein
HAT: histone acetyltransferase
HDAC: histone deacetylase
Hprt: hypoxanthine-guanine phosphoribosyltransferase
LDH: lactate dehydrogenase
PBS: phosphate-buffered saline
qPCR: quantitative polymerase chain reaction
RT: room temperature
RXR: retinoid X receptor
TBT: tributyltin
TPT: triphenyltin
UV: ultraviolet light

## References

[1] Danovaro R, Bongiorni L, Corinaldesi C, Giovannelli D, Damiani E, Astolfi P, Greci L, Pusceddu A (2008). Sunscreens cause coral bleaching by promoting viral infections. Environ Health Perspect.

[2] Zhang T, Sun H, Kannan K (2013). Blood and urinary bisphenol A concentrations in children, adults, and pregnant women from China: partitioning between blood and urine and maternal and fetal cord blood. Environ Sci Technol.

[3] Calafat AM, Wong LY, Ye X, Reidy JA, Needham LL (2008). Concentrations of the sunscreen agent benzophenone-3 in residents of the United States. Environ Health Perspect.

[4] Philippat C, Mortamais M, Chevrier C, Petit C, Calafat AM, Ye X, Silva MJ, Brambilla C, Pin I, Charles MA, Cordier S, Slama R (2012). Exposure to phthalates and phenols during pregnancy and offspring size at birth. Environ Health Perspect.

[5] Wolff MS, Engel SM, Berkowitz GS, Ye X, Silva MJ, Zhu C, Wetmur J, Calafat AM (2008). Prenatal phenol and phthalate exposures and birth outcomes. Environ Health Perspect.

[6] Frederiksen H, Jensen TK, Jørgensen N, Kyhl HB, Husby S, Skakkebæk NE, Main KM, Juul A, Andersson AM (2014). Human urinary excretion of non-persistent environmental chemicals: an overview of Danish data collected between 2006 and 2012. Reproduction.

[7] Frederiksen H, Nielsen JK, Mørck TA, Hansen PW, Jensen JF, Nielsen O, Andersson AM, Knudsen LE (2013). Urinary excretion of phthalate metabolites, phenols and parabens in rural and urban Danish mother-child pairs. Int J Hyg Environ Health.

[8] Janjua NR, Kongshoj B, Andersson AM, Wulf HC (2008). Sunscreens in human plasma and urine after repeated whole-body topical application. J Eur Acad Dermatol Venereol.

[9] Jiang R, Roberts MS, Collins DM, Benson HA (1999). Absorption of sunscreens across human skin: an evaluation of commercial products for children and adults. Br J Clin Pharmacol.

[10] Schlumpf M, Kypke K, Wittassek M, Angerer J, Mascher H, Mascher D, Vökt C, Birchler M, Lichtensteiger W (2010). Exposure patterns of UV-filters, fragrances, parabens, phthalates, organochlor pesticides, PBDEs, and PCBs in human milk: correlation of UV-filters with use of cosmetics. Chemosphere.

[11] Huo W, Cai P, Chen M, Li H, Tang J, Xu C, Zhu D, Tang W, Xia Y (2016). The relationship between prenatal exposure to BP-3 and Hirschsprung's disease. Chemosphere.

[12] Broniowska Ż, Pomierny B, Smaga I, Filip M, Budziszewska B (2016). The effect of UV-filters on the viability of neuroblastoma (SH-SY5Y) cell line. Neurotoxicology May.

[13] Wnuk A, Rzemieniec J, Lasoń W, Krzeptowski W, Kajta M (2017) Apoptosis induced by the UV filter benzophenone-3 in mouse neuronal cells is mediated via attenuation of Erα/Pparγ and stimulation of Erβ/Gpr30 signaling. Mol Neurobiol. doi:10.1007/s12035-017-0480-z 10.1007/s12035-017-0480-z PMC5840254 28357806

[14] Ma R, Cotton B, Lichtensteiger W, Schlumpf M (2003). UV filters with antagonistic action at androgen receptors in the MDA-kb2 cell transcriptional-activation assay. Toxicol Sci.

[15] Schlumpf M, Cotton B, Conscience M, Haller V, Steinmann B, Lichtensteiger W (2001). In vitro and in vivo estrogenicity of UV screens. Environ Health Perspect.

[16] Schreurs RH, Sonneveld E, Jansen JH, Seinen W, van der Burg B (2005). Interaction of polycyclic musks and UV-filters with the estrogen receptor (ER), androgen receptor (AR), and progesterone receptor (PR) in reporter gene bioassays. Toxicol Sci.

[17] Kunisue T, Chen Z, Buck Louis GM, Sundaram R, Hediger ML, Sun L, Kannan K (2012). Urinary concentrations of benzophenone-type UV filters in US women and their association with endometriosis. Environ Sci Technol.

[18] Evans, R.M., Mangelsdorf, D.J. (2014) Nuclear receptors, RXR, and the big bang. Cell 2014;157(1):255–266. doi: 10.1016/j.cell.2014.03.012 10.1016/j.cell.2014.03.012 PMC4029515 24679540

[19] Rőszer T, Menéndez-Gutiérrez MP, Cedenilla M, Ricote M (2013). Retinoid X receptors in macrophage biology. Trends Endocrinol Metab.

[20] Zhang H, Chen L, Chen J, Jiang H, Shen X (2011). Structural basis for retinoic X receptor repression on the tetramer. J Biol Chem.

[21] Huang JK, Jarjour AA, Nait Oumesmar B, Kerninon C, Williams A, Krezel W, Kagechika H, Bauer J, Zhao C, Baron-Van Evercooren A, Chambon P, Ffrench-Constant C, Franklin RJ (2011). Retinoid X receptor gamma signaling accelerates CNS remyelination. Nat Neurosci.

[22] Wnuk A, Rzemieniec J, Litwa E, Lasoń W, Krzeptowski W, Wójtowicz AK, Kajta M (2016). The crucial involvement of retinoid X receptors in DDE neurotoxicity. Neurotox Res.

[23] Litwa E, Rzemieniec J, Wnuk A, Lason W, Krzeptowski W, Kajta M (2014). Apoptotic and neurotoxic actions of 4-para-nonylphenol are accompanied by activation of retinoid X receptor and impairment of classical estrogen receptor signaling. J Steroid Biochem Mol Biol.

[24] Litwa E, Rzemieniec J, Wnuk A, Lasoń W, Krzeptowski W, Kajta M (2016). RXRα, PXR and CAR xenobiotic receptors mediate the apoptotic and neurotoxic actions of nonylphenol in mouse hippocampal cells. J Steroid Biochem Mol Biol.

[25] Shutoh Y, Takeda M, Ohtsuka R, Haishima A, Yamaguchi S, Fujie H, Komatsu Y, Maita K, Harada T (2009). Low dose effects of dichlorodiphenyltrichloroethane (DDT) on gene transcription and DNA methylation in the hypothalamus of young male rats: implication of hormesis-like effects. J Toxicol Sci.

[26] Huen K, Yousefi P, Bradman A, Yan L, Harley KG, Kogut K, Eskenazi B, Holland N (2014). Effects of age, sex, and persistent organic pollutants on DNA methylation in children. Environ Mol Mutagen.

[27] Volmar CH, Wahlestedt C (2015). Histone deacetylases (HDACs) and brain function. Neuroepigenetics Volume.

[28] Glick D, Barth S, Macleod KF (2010). Autophagy: cellular and molecular mechanisms. J Pathol.

[29] Kajta M, Domin H, Grynkiewicz G, Lasoń W (2007). Genistein inhibits glutamate-induced apoptotic processes in primary neuronal cell cultures: an involvement of aryl hydrocarbon receptor and estrogen receptor/glycogen synthase kinase-3beta intracellular signaling pathway. Neuroscience.

[30] Kajta M, Makarewicz D, Ziemińska E, Jantas D, Domin H, Lasoń W, Kutner A, Łazarewicz JW (2009). Neuroprotection by co-treatment and post-treating with calcitriol following the ischemic and excitotoxic insult in vivo and in vitro. Neurochem Int.

[31] Nicholson DW, Ali A, Thornberry NA, Vaillancourt JP, Ding CK, Gallant M, Gareau Y, Griffin PR, Labelle M, Lazebnik YA, Munday NA, Raju SM, Smulson ME, Yamin T-T, Yu VL, Miller DK (1995). Identification and inhibition of the ICE/CED-3 protease necessary for mammalian apoptosis. Nature.

[32] Kajta M, Trotter A, Lasoń W, Beyer C (2005). Effect of NMDA on staurosporine-induced activation of caspase-3 and LDH release in mouse neocortical and hippocampal cells. Brain Res Dev Brain Res.

[33] Wójtowicz AK, Kajta M, Gregoraszczuk EŁ (2007). DDT- and DDE-induced disruption of ovarian steroidogenesis in prepubertal porcine ovarian follicles: a possible interaction with the main steroidogenic enzymes and estrogen receptor beta. J Physiol Pharmacol.

[34] Rzemieniec J, Litwa E, Wnuk A, Lasoń W, Gołas A. Krzeptowski W, Kajta M (2015) Neuroprotective action of raloxifene against hypoxia-induced damage in mouse hippocampal cells depends on ERα but not ERβ or GPR30 signalling. J steroid Biochem Mol Biol. 146:26-37. doi: 10.1016/j.jsbmb.2014.05.005. 10.1016/j.jsbmb.2014.05.005 24846829

[35] Rzemieniec J, Litwa E, Wnuk A, Lason W, Krzeptowski W, Kajta M (2016). Selective aryl hydrocarbon receptor modulator 3,3′-diindolylmethane impairs AhR and ARNT signaling and protects mouse neuronal cells against hypoxia. Mol Neurobiol.

[36] Kajta M, Rzemieniec J, Litwa E, Lasoń W, Lenartowicz M, Krzeptowski W, Wójtowicz AK (2013). The key involvement of estrogen receptor β and G-protein-coupled receptor 30 in the neuroprotective action of daidzein. Neuroscience.

[37] Wang L, Asimakopoulos AG, Kannan K (2015). Accumulation of 19 environmental phenolic and xenobiotic heterocyclic aromatic compounds in human adipose tissue. Environ Int.

[38] Schlecht C, Klammer H, Jarry H, Wuttke W (2004). Effects of estradiol, benzophenone-2 and benzophenone-3 on the expression pattern of the estrogen receptors (ER) alpha and beta, the estrogen receptor-related receptor 1 (ERR1) and the aryl hydrocarbon receptor (AhR) in adult ovariectomized rats. Toxicology.

[39] Zucchi S, Blüthgen N, Ieronimo A, Fent K (2010). The UV-absorber benzophenone-4 alters transcripts of genes involved in hormonal pathways in zebrafish (Danio rerio) eleuthero-embryos and adult males. Toxicol Appl Pharmacol.

[40] Ayala-Peña VB, Pilotti F, Volonté Y, Rotstein NP, Politi LE, German OL (2016). Protective effects of retinoid x receptors on retina pigment epithelium cells. Biochim Biophys Acta.

[41] Papi A, Govoni M, Ciavarella C, Spisni E, Orlandi M, Farabegoli F (2016). Epigallocatechin-3-gallate increases RXRγ-mediated pro-apoptotic and anti-invasive effects in gastrointestinal cancer cell lines. Curr Cancer Drug Targets.

[42] Qin S, Okawa Y, Atangan LI, Brown G, Chandraratna RA, Zhao Y (2008). Integrities of A/B and C domains of RXR are required for rexinoid-induced caspase activations and apoptosis. J Steroid Biochem Mol Biol.

[43] Akram A, Schmeidler J, Katsel P, Hof PR, Haroutunian V (2010). Increased expression of RXRα in dementia: an early harbinger for the cholesterol dyshomeostasis?. Mol Neurodegener.

[44] Wietrzych M, Meziane H, Sutter A, Ghyselinck N, Chapman PF, Chambon P, Krezel W (2005). Working memory deficits in retinoid X receptor gamma-deficient mice. Learn Mem.

[45] Wang X, Liu J, Long Z, Sun Q, Liu Y, Wang L, Zhang X, Hai C (2015). Effect of diosgenin on metabolic dysfunction: role of ERβ in the regulation of PPARγ. Toxicol Appl Pharmacol.

[46] Lefebvre P, Benomar Y, Staels B (2010). Retinoid X receptors: common heterodimerization partners with distinct functions. Trends Endocrinol Metab.

[47] Kajta M, Wnuk A, Rzemieniec J, Litwa E, Lason W, Zelek-Molik A, Nalepa I, Rogóż Z et al (2017) Depressive-like effect of prenatal exposure to DDT involves global DNA hypomethylation and impairment of GPER1/ESR1 protein levels but not ESR2 and AHR/ARNT signaling. J Steroid Biochem Mol Biol. doi:10.1016/j.jsbmb.2017.03.001 10.1016/j.jsbmb.2017.03.001 28263910

[48] Osada S, Nishikawa J, Nakanishi T, Tanaka K, Nishihara T (2005). Some organotin compounds enhance histone acetyltransferase activity. Toxicol Lett.

